# Tailings facility disclosures reveal stability risks

**DOI:** 10.1038/s41598-021-84897-0

**Published:** 2021-03-05

**Authors:** Daniel M. Franks, Martin Stringer, Luis A. Torres-Cruz, Elaine Baker, Rick Valenta, Kristina Thygesen, Adam Matthews, John Howchin, Stephen Barrie

**Affiliations:** 1 Sustainable Minerals Institute, The University of Queensland, Brisbane, QLD 4072 Australia; 2 W.H. Bryan Mining and Geology Research Centre, Sustainable Minerals Institute, The University of Queensland, 40 Isles Road Indooroopilly, Brisbane, QLD 4068 Australia; 3 School of Civil and Environmental Engineering, University of the Witwatersrand, 1 Jan Smuts Avenue, Braamfontein, 2000 Johannesburg South Africa; 4 UNESCO Chair in Marine Science, The University of Sydney, Madsen FO9, Sydney, NSW 2006 Australia; 5 GRID Arendal, P.O. Box 183, 4802 Arendal, Norway; 6 Investor Mining and Tailings Safety Initiative and Church of England Pensions Board, Church House, Great Smith Street, London, SW1P 3AZ UK; 7 Investor Mining and Tailings Safety Initiative and Council On Ethics for the Swedish National Pension Funds, Gothenburg, Sweden

**Keywords:** Environmental impact, Natural hazards, Civil engineering, Geology

## Abstract

Tailings facility failures represent a significant risk to the environment and communities globally, but until now little data was available on the global distribution of risks and characteristics of facilities to ensure proper governance. We conducted a survey and compiled a database with information on tailings facilities disclosed by extractive companies at the request of institutional investors. Despite limitations in the data, this information disclosure request represents the most comprehensive survey of tailings facilities ever undertaken. The compiled dataset includes 1743 tailings facilities and provides insights into a range of topics including construction method, stability, consequence of failure, stored volume, and the rate of uptake of alternative technologies to dewater tailings and reduce geotechnical risk. Our analysis reveals that 10 per cent of tailings facilities reported notable stability concerns or failure to be confirmed or certified as stable at some point in their history, with distinct trends according to construction method, governance, age, height, volume and seismic hazard. Controversy has surrounded the safety of tailings facilities, most notably upstream facilities, for many years but in the absence of definitive empirical data differentiating the risks of different facility types, upstream facilities have continued to be used widely by the industry and a consensus has emerged that upstream facilities can theoretically be built safely under the right circumstances. Our findings reveal that in practice active upstream facilities report a higher incidence of stability issues (18.3%) than other facility types, and that this elevated risk persists even when these facilities are built in high governance settings. In-pit/natural landform and dry-stack facilities report lower incidence of stability issues, though the rate of stability issues is significant by engineering standards (> 2 per cent) across all construction methods, highlighting the universal importance of careful facility management and governance. The insights reported here can assist the global governance of tailings facility stability risks.

## Introduction

Tailings facility failures can leave serious environmental, social and economic legacies^1^. They have disproportionately shaped the reputation of the minerals industry, eroded public trust, and are reshaping the risk calculations made by financial institutions and investors^2^. On the 25th of January 2019, Tailings Dam 1 at the Córrego do Feijão iron ore mine, near the town of Brumadinho, in Minas Gerais, Brazil, failed catastrophically causing 259 deaths, with a further 11 people reported missing, as well as widespread damage to mine facilities, and downstream ecosystems^3^. The disaster shortly followed two other high-profile tailings dam failures, one at the Fundão tailings facility, Brazil, in 2015, and another at the Mount Polley tailings facility, Canada, in 2014. The series of failures triggered the creation of the Investor Mining and Tailings Safety Initiative, a group of 112 institutional investors that represent US$14 trillion in assets under management. On April 5, 2019, this group of investors issued a tailings information disclosure request that ultimately reached 726 publicly-listed extraction companies^4^. The requested information covered 20 aspects of each tailings facility, including coordinates, size, construction method, and history (Table S1). As requested by the investors, the 107 companies that disclosed information posted their disclosures on their websites. We compiled these disclosures and enhanced the dataset with information regarding seismicity, commodity production, wind speed, and precipitation (data sources in SM). The resulting database, which constitutes the most comprehensive global repository of information on tailings facilities, is available for download as a supplementary file (Data S1).

The database includes tailings facilities owned by publicly-listed companies and excludes facilities that are abandoned, state-owned, and privately-owned. As a consequence, the database probably over-represents larger facilities (see SM for details). Despite these limitations, the release of this information to the public domain and its compilation into a single database constitutes a major step towards better managing the risk posed by tailings. Until now our understanding of the global distribution of risks and characteristics of tailings facilities has been based on analyses of individual tailings facility failures^5–7^ and datasets of multiple tailings facility failures^8–10^. However, to date, there is no global public inventory of tailings facilities^1^. Here we report on the major findings from analysis of this newly disclosed information on global tailings storage.

## Results

### Tailings storage

**Figure 1:**
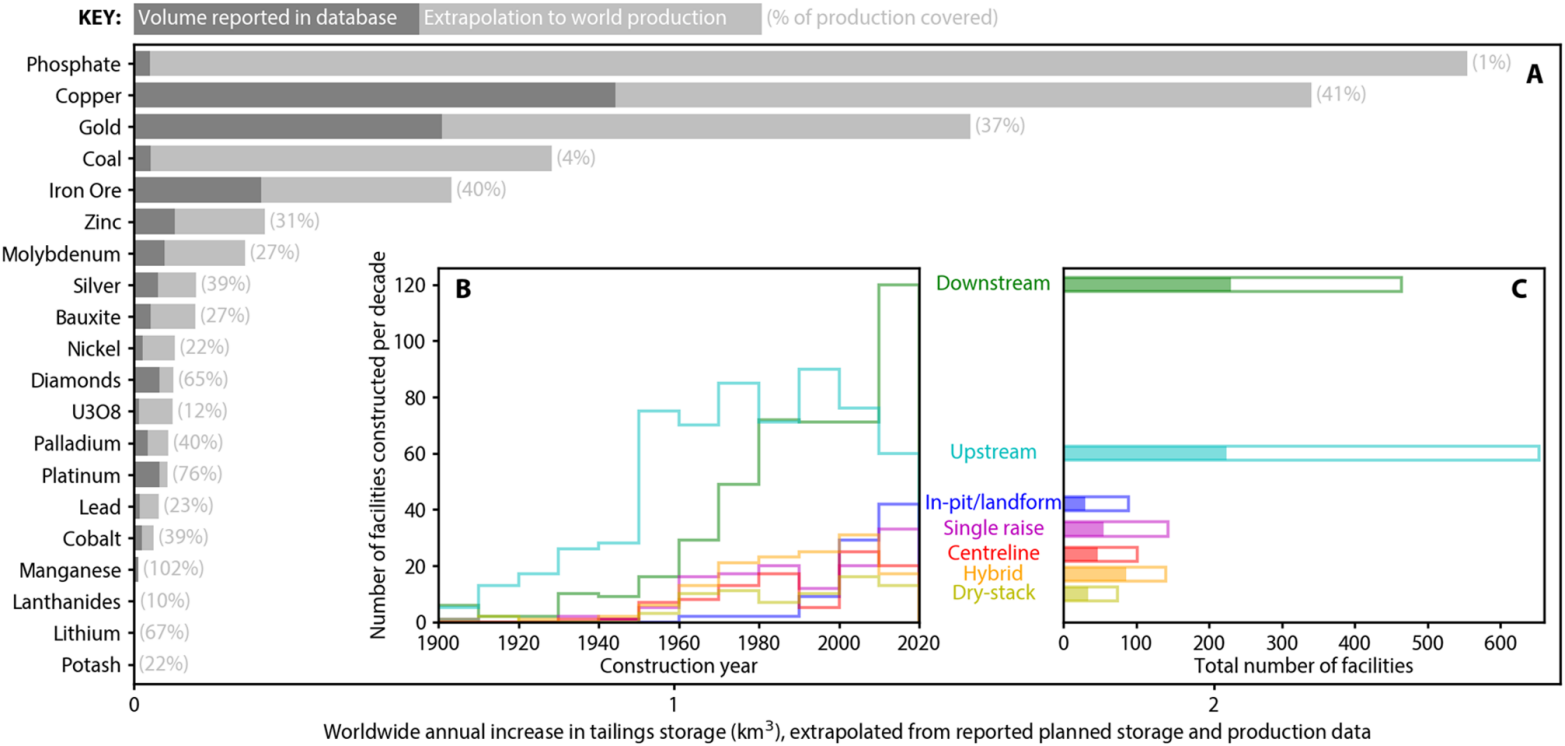
Tailings storage. (**A**) Tailings storage volume increase per year over the 2019–2023 period for a range of commodities as reported in the dataset and extrapolated to world production. (**B**) Number of reported facilities constructed per decade by raise type. (**C**) Reported tailings facilities by raise type. Shading indicates active facilities.

The survey of tailings facilities revealed data on 1743 facilities (725 of which are currently active), representing an average of 36 per cent of contemporary global commodity production (Fig. 1A). A total of 44.5 billion m^3^ of tailings are currently stored by the facilities disclosed in the dataset. For a sense of scale, if this volume were spread evenly across an area the size of Manhattan Island, it would be 750 m high, which is 200 m taller than the tallest skyscraper. The mean facility volume for all facilities is 26.3 million m^3^ and for active facilities it is 43.7 million m^3^. The volume of tailings under storage varies with facility construction method. Upstream facilities contain the highest total volume of tailings under storage, followed by downstream, hybrid, centreline, single raise, in-pit/natural landform, dry-stack and other (Fig. S1). The term “hybrid” facility is used here to refer to facilities where multiple raise methods are utilised in the same facility over time. The highest median volume of tailings stored per facility corresponds to hybrid facilities (18.3 million m^3^), followed by centreline (7.3 million m^3^) and upstream (5.9 million m^3^).

Expected generation of tailings over the coming five years is 2.5 billion m^3^ per year for the reporting companies (2019–2023). This represents a 26 per cent increase in tailings under storage over this five-year period to 56.2 billion m^3^ at January 2024. That is, the rate of tailings production is accelerating. To estimate the total number of tailings facilities worldwide, and the expected global generation of tailings from all facilities, we proportionally extrapolated the data from the disclosures to account for global mineral commodity production estimated by the United States Geological Survey (USGS) (Fig. 1A).

This extrapolation implies that 10 billion m^3^ (~ 13 billion *t*) of additional tailings per year will require storage in existing or planned facilities over the coming five-year period. This estimate excludes tailings that are not stored in a tailings facility (e.g. tailings backfill and heap leach pads). We estimate that the total number of active tailings facilities worldwide is around 3,400 (see Table S2). If the proportion of active, inactive, and closed facilities in the database is assumed to be similar for facilities that are not in the database, a lower bound for the total number of facilities (active, inactive and closed) could be estimated at around 8,100. This estimate does not include abandoned facilities and should be considered a conservative estimate due to the fact that companies responsible for inactive and closed facilities are likely to be under-represented in the survey responses. Previous authors have cited global estimates of 3500 tailings facilities^11^ and 12,000 facilities (just in China^12^). However, the methods for determining the aforementioned estimates are not stated, and it is not clear whether they refer to active, inactive, closed, or abandoned facilities.

The number of tailings facilities doubled between 1955 and 1969 (14 years), doubled again between 1969 and 1989 (20 years) and again between 1989 and 2020 (31 years; Fig. S2). This increase in doubling time, coupled with the accelerating rate in tailings production noted above, indicates that the tailings storage facilities are, on average, increasing in size.

The extent to which the mining industry has adopted different construction methods has evolved with time. For instance, while upstream facilities make up 37 per cent of the total, they have declined from a peak of 85 per cent of new facilities in 1920–1929 to 19 per cent of new facilities in 2010–2019 (Fig. 1B). When all facilities, active or otherwise, are considered, upstream construction, followed by downstream construction are the most common methods (Fig. 1C). Centreline, hybrid, and single raise construction methods are the next most common. In-pit/natural landform, and dry-stacked are the least common facility types. Owing to their historical popularity, upstream facilities make-up 43 per cent of facilities that are inactive, closed or reclaimed. However, in the past twenty years, the number of new downstream and in-pit/natural landform facilities have risen sharply (Fig. 1B). At present, the number of active downstream facilities (230) marginally exceeds the number of active upstream facilities (224) (Fig. 1C).

The relative frequency of facility construction methods varies by continent (Fig. S3). This is due to a range of factors, including commodity, ore type, climate, seismic hazard, topography, and governance. Upstream facilities represent a relatively low number of active facilities in North and South America when compared to Africa and Oceania. This is partly explained by different regulatory approaches; for example, upstream facilities were banned in Chile in 1970, in Peru in 2014 and in Brazil in 2019, but is also likely a function of climatic and topographic factors. The ban in Chile, and concerns over the relative stability of upstream facilities in locations with elevated seismic hazard, may be reasons for the lower proportion of upstream facilities observed with increasing seismic hazard (Fig. S4).

The removal of water from tailings is an important innovation with the potential to improve geotechnical and geochemical stability^2,13–18^. Dewatering technologies have experienced a wave of different advances over the past few decades: cycloning in the late 1960s, thickening in the mid-1970s, filtration in the 1980s and paste facilities from the 1990s^17^. Unfortunately, the disclosures do not differentiate paste and thickened tailings from conventional wet tailings. However, the disclosures identify dry-stack facilities, which are facilities that employ a type of dewatering scheme. This category includes both in-situ dewatering of tailings (sometimes referred to as mud-farming) and the filtering of tailings prior to deposition (beginning in the 1980s).

Although mining companies and peak industry bodies have identified the adoption of dewatered tailings as a priority^1^, the data indicate that only 13 dry-stack facilities were constructed in the last decade. Furthermore, since 1970, the percentage of new tailings facilities that are dry-stack has remained stagnant between 3 and 6% (Fig. S5). This finding is further confirmed by the fact that just one international mining company operates, or is the majority shareholder in, 53 of the 74 (72%) dry-stack facilities in the dataset. This raises a question about whether the economic and policy incentives to transition to these newer technologies are sufficient, noting that dry-stack facilities are typically associated with higher operating costs and lower production capacity^17,19^.

### Stability of tailings facilities

**Figure 2:**
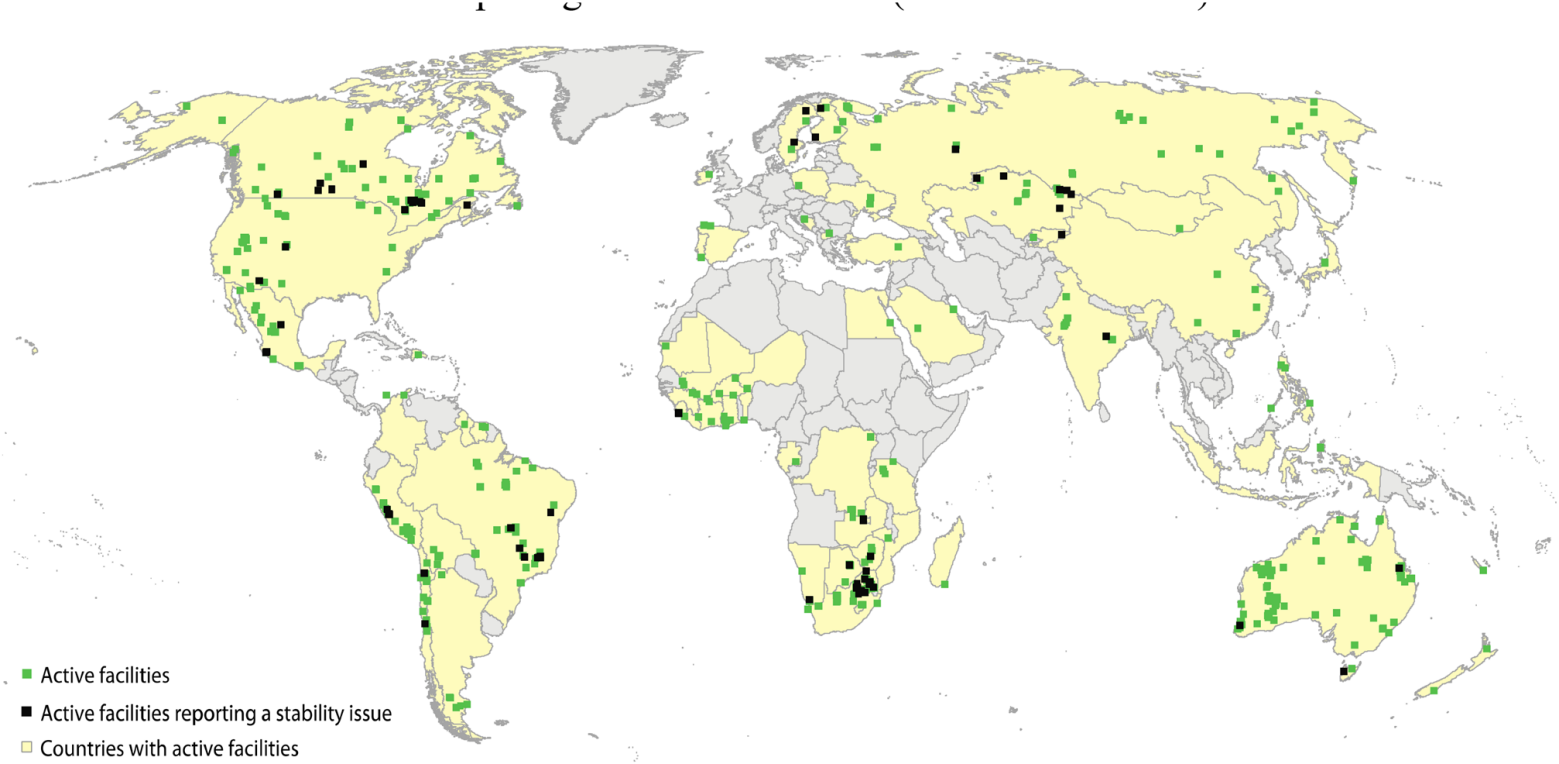
Map of active tailings facilities. Location of active facilities and active facilities reporting a stability issue. Shaded countries indicate those where an active tailings facility is reported in the dataset. The map illustrates the wide geographic representation of the surveyed facilities, as well as the under-representation of facilities in countries such as China, India and Chile, where a higher proportion of companies are not publicly-listed.

Companies were requested to disclose any situation where a facility, “at any point in its history, failed to be confirmed or certified as stable, or experienced notable stability concerns, as identified by an independent engineer (even if later certified as stable by the same or a different firm).” The reported issues ranged in seriousness from relatively minor to very serious. Figure 2 shows the location of active tailings facilities reporting a stability issue. In total, 10 per cent of all facilities reported a stability issue. The proportion of facilities reporting a stability issue increases with age (Fig. S6), highlighting the importance of closely monitoring legacy and closed facilities. Reported stability issues also increase as the facilities get taller, up to a height of 100 m. Thereafter, the proportion gets smaller. In fact, none of the 27 facilities taller than 140 m reported a stability issue (Fig. S7). This may be a consequence of the greater engineering expertise called upon to develop these tall structures, and of their relatively younger age. Stability issues increase steadily with stored volume, with 20% of the facilities with volumes exceeding 100 Mm^3^ reporting stability issues (Fig. S8). The effect of seismic hazard is complex and somewhat counterintuitive. The prevalence of stability issues decreases from 19 to 3 per cent as seismic hazard changes from low to moderate, but increases to 21 per cent as seismic hazard goes from moderate to high and very high (Fig. S9). Fourteen per cent of the tailings facilities are exposed to high or very high seismic hazard based on estimates of peak ground acceleration (see SM for details).

Tailings facilities located in OECD-countries, as well as those operated by ICMM-member companies generally reported a lower incidence of stability issues (see Table S3). This lends some weight to the view that tailings governance plays a role in ensuring geotechnical stability. However, the incidence of stability issues for facilities located in OECD-countries or operated by ICMM-member companies, remains high (~ 10 to 20%) across a number of raise types (most notably upstream, hybrid and centreline).

**Figure 3:**
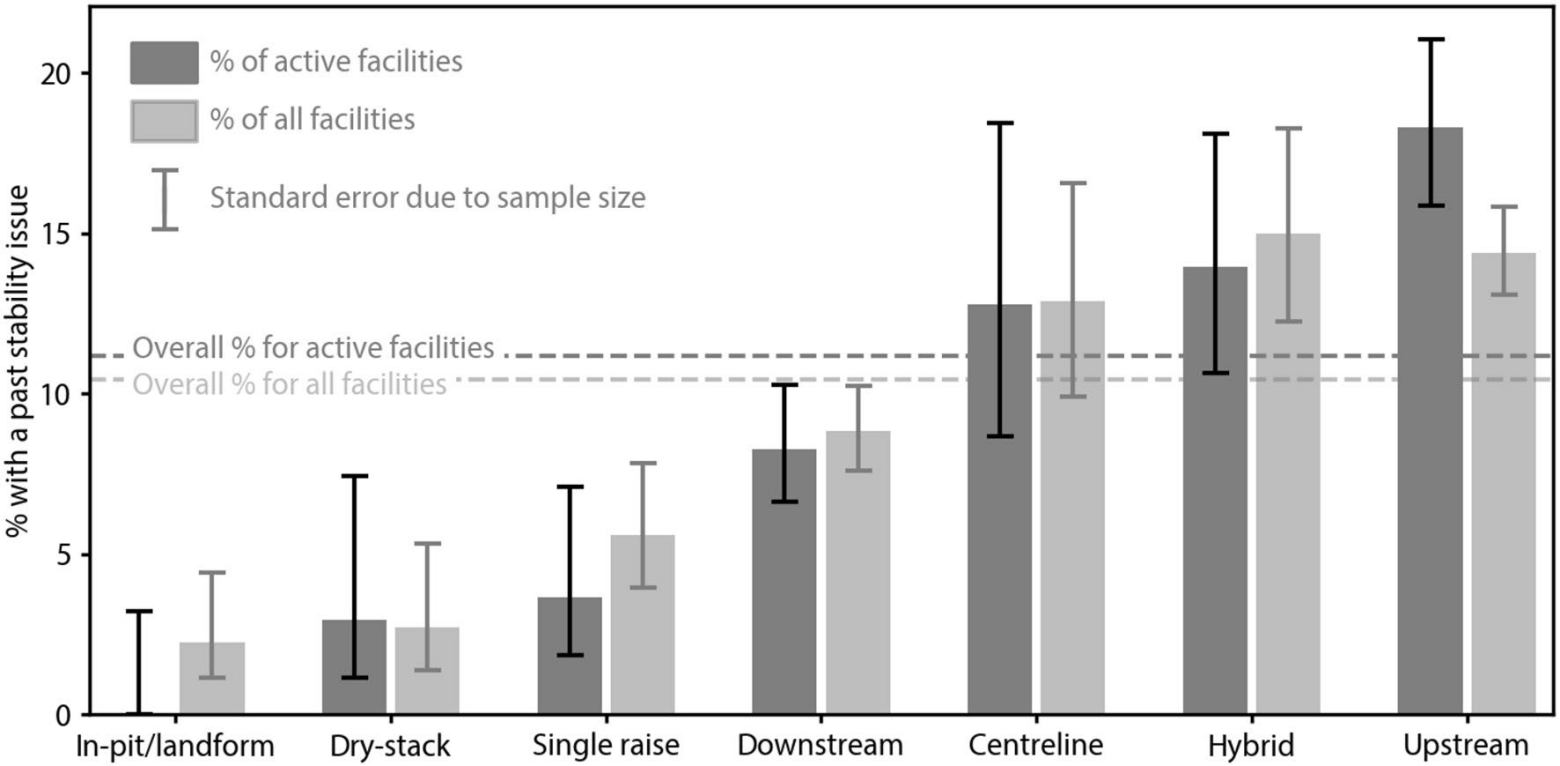
Stability of tailings facilities. Proportion of facilities with a stability issue by raise type. Error bar lengths are binomial confidence intervals for the subsample represented by each bar, showing ± 1 standard error (approximately 68%).

Upstream and hybrid facilities were the most likely to report a stability issue. They were followed by centreline, downstream and single raise facilities (Fig. 3). The likelihood of a stability issue in active upstream facilities is twice that of active downstream facilities and six times as many that of active dry-stack facilities. No active in-pit/natural landform facilities reported a stability issue. More generally, the rate of stability issues is significant by engineering standards (> 2 per cent) across all construction methods, highlighting the universal importance of careful facility management and governance.

Interpretation of the results shown in Fig. 3 should take into account both the uncertainties shown, and that raise type is not the only difference between the subsamples; their distribution also varies in terms of other parameters that may affect geotechnical stability. To estimate this effect, we controlled for six parameters: age, height, volume, seismic hazard, wind speed, and rainfall (Fig. S10 & S11).

Considering the category with the highest incidence, Fig. 3 shows that active upstream facilities were the most likely to report a stability issue, with the incidence a few standard errors above that for the dataset as a whole. The control tests showed that the properties of the upstream samples (notably their distribution of age), have a small effect on the incidence of stability, however the estimated effect is only about one standard error, and is not sufficient to account for their higher than average incidence. At the lowest incidence, the very low in-pit/landform percentage is also partly accounted for by sample distribution across the control parameters, but again the estimated effect is not sufficient to explain the very significant deviance from the overall mean. The estimated effects of sample distribution on the dry-stack samples were very small and tended to increase their incidence.

### Consequences of facility failure

The consequence of failure category for each tailings facility was reported by the companies. Consequence ratings are typically classified as part of modelling undertaken in the facility design phase and are independent of the likelihood of failure of the facility. The categories correspond to various country-level, industry and corporate classification systems, using different metrics of consequence. Tailings facilities were classified against a total of 62 different classification schemes. The five most common schemes reported in the dataset are listed in Table S4. Collectively these schemes cover 68 per cent of all facilities and 76 per cent of currently active facilities.

**Figure 4:**
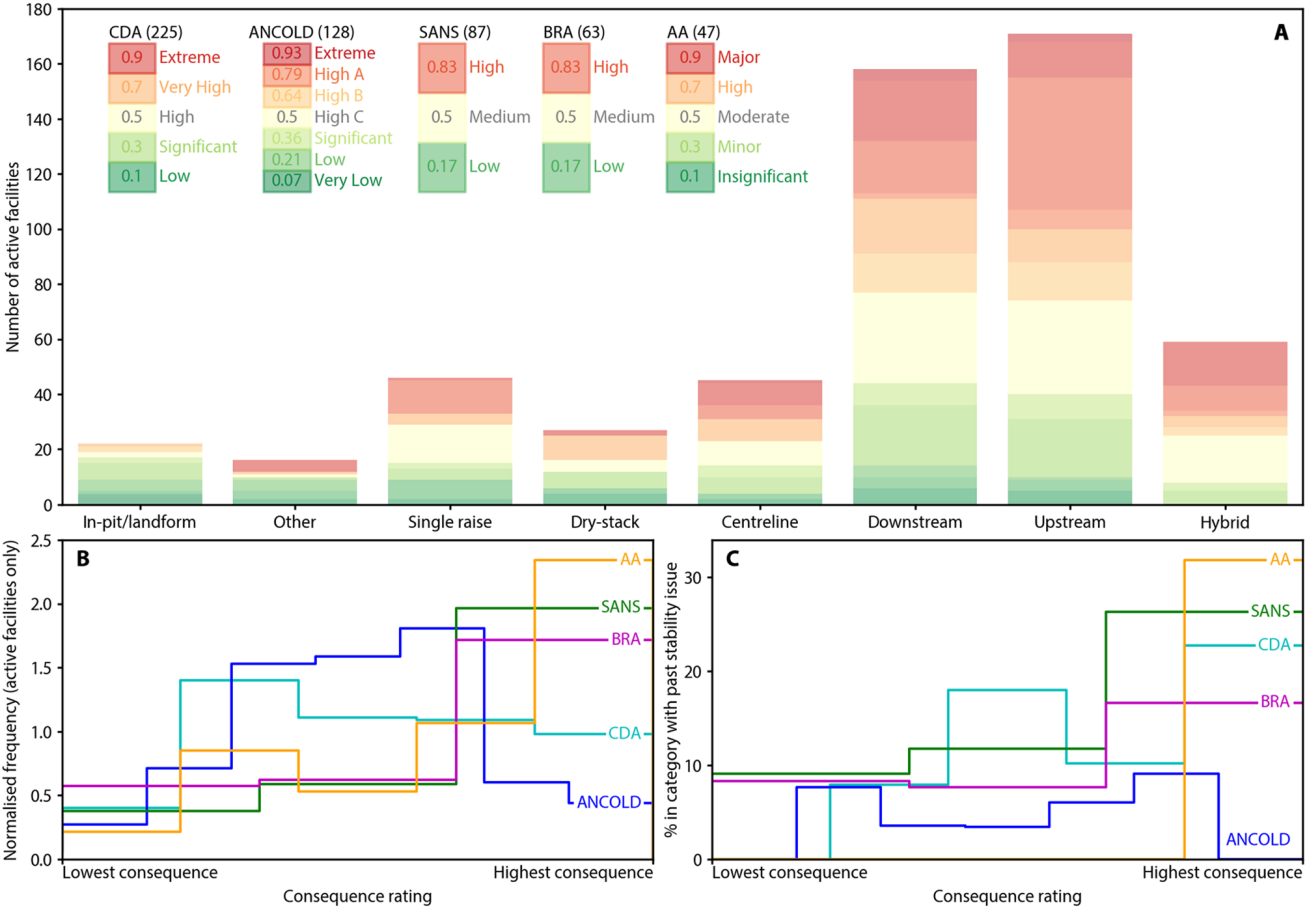
Consequences of facility failure. (**A**) Consequence of failure by facility raise type (active facilities) for the five most common consequence classification schemes (*CDA* Canadian Dam Association, *ANCOLD* Australian National Committee on Large Dams, *SANS* South African National Standards, *BRA* Brazilian Ordinance 70.389/17, *AA* Anglo American Technical Standard). (**B**) Distribution of active facilities by consequence rating for the five most common consequence classification schemes. To allow comparison of the distributions, the frequency of the Y-axis is normalised so that the area under each consequence classification curve is the same. (**C**) Proportion of sites reporting a stability issue by consequence of failure.

Figure 4A reports consequence of failure by facility raise type for active facilities across the five most common schemes. A trend is apparent where conventional facilities (i.e. hybrid, upstream, downstream and centerline) are more likely to be associated with higher consequence ratings than are the remaining raise types. This general trend holds across each of the individual consequence schemes and is at least partly explained by the nature of tailings flow. That is, tailings that are hydraulically deposited in conventional facilities have higher flow potential due to their greater water content. A significant number of facilities (501; 29%) have not formally considered the downstream effects of a hypothetical catastrophic failure, 364 of the 501 (73%) are facilities that are not active.

Given that upstream facilities have been considered by ICOLD and UNEP^8^ to be less safe than downstream and centreline facilities, it could be expected that upstream construction would be avoided in locations where the potential consequence of failure is high. Notwithstanding the fact that upstream construction has been banned in Chile, and more recently, Peru and Brazil, this does not appear to be the case.

Figure 4B shows the frequency of active facilities by consequence category for each of the five most common schemes. For the Anglo American Technical Standard (AA), South African National Standards (SANS) and Brazilian Ordinance 70.389/17 (BRA) schemes, a trend is apparent where the number of facilities increases progressively with increasing consequence rating.

Figure 4C illustrates the likelihood of a stability issue within each consequence category for the five most common schemes. A trend is apparent across most schemes (with the exception of the Australian National Committee on Large Dams scheme) where facilities with a higher consequence rating are more likely to have reported a stability issue. This finding is somewhat counterintuitive as higher consequence facilities are built to higher construction standards. This finding may in part be explained by the lower proportion of dry-stack and in-pit/natural landform facilities that are classified in higher consequence categories, which are also associated with a lower likelihood of stability issues.

## Outlook

The dataset compiled and analysed herein constitutes an important first step towards creating greater accountability from the mining industry to the public in general. For instance, for the first time ever, the coordinates of 1743 tailings storage facilities are in the public domain. As illustrated by Figures S4, S9-S11, this enables persons outside of the mining companies to use remote sensing tools or geographic information systems to better understand the risks to which these facilities are exposed.

Although a solid step in the right direction, there is still plenty to improve in the way in which mining companies disclose information about their tailings facilities. For instance, our analysis showed that the current database accounts, on average, for 30% of different types of commodity production. Clearly there is a need to move closer to 100% coverage. Investors are continuing to engage with non-responsive companies but their focus is on publicly-listed companies. Engagement with privately-owned and state-owned companies is also necessary to improve coverage. Record keeping also appears to be a significant issue, with companies unable to report complete engineering records for 257 of the 1743 facilities (15%), the majority of which are facilities that are no longer active (195 of 257; 76%). Furthermore, the lack of a centralized platform that ensures a standardised capture of the disclosures resulted in a wide variability in the quality of the answers. It is hoped that there will be an opportunity to implement such a centralised platform in future rounds of disclosure, which should ideally be the norm. We expect that the investor disclosure request and company disclosures will develop over time, improving the dataset available for analysis, and we believe that this will be a fruitful area of collaboration between mining companies, academics, investors, regulators, civil society, and multilateral agencies. Greater disclosure is also being requested at the country-level, with the Chilean Transparent Tailings Initiative^20^, requiring permanent, online disclosure of information on 104 active tailings facilities. Together these initiatives point to a greater appetite by investors and governments for transparency on the characteristics and risks of tailings facilities.

The sheer scale of global tailings production and the high impact of tailings facility failures highlights the need to improve all aspects of tailings disposal and management. Furthermore, the data highlight the need to continue developing management options and technologies to both minimize tailings production and to repurpose tailings to reduce storage requirements and their associated risks. In the short term, the data suggest that ever larger tailings storage facilities will continue to be built in locations with ever higher consequences of failure. Greater transparency brought about by these and future disclosures could play an important role in the reduction, and ideally elimination, of catastrophic tailings facility failures.

## Supplementary Information

Supplementary Information 1.

Supplementary Information 2.

## Data Availability

All data are available in the manuscript, the supplementary materials, or at repositories that are either publicly accessible, or accessible with a subscription (S&P Global).
