# Author Correction: Tailings facility disclosures reveal stability risks

**DOI:** 10.1038/s41598-021-91384-z

**Published:** 2021-06-03

**Authors:** Daniel M. Franks, Martin Stringer, Luis A. Torres-Cruz, Elaine Baker, Rick Valenta, Kristina Thygesen, Adam Matthews, John Howchin, Stephen Barrie

**Affiliations:** 1 Sustainable Minerals Institute, The University of Queensland, Brisbane, QLD 4072 Australia; 2 W.H. Bryan Mining and Geology Research Centre, Sustainable Minerals Institute, The University of Queensland, 40 Isles Road Indooroopilly, Brisbane, QLD 4068 Australia; 3 School of Civil and Environmental Engineering, University of the Witwatersrand, 1 Jan Smuts Avenue, Braamfontein, 2000 Johannesburg South Africa; 4 UNESCO Chair in Marine Science, The University of Sydney, Madsen FO9, Sydney, NSW 2006 Australia; 5 GRID Arendal, P.O. Box 183, 4802 Arendal, Norway; 6 Investor Mining and Tailings Safety Initiative and Church of England Pensions Board, Church House, Great Smith Street, London, SW1P 3AZ UK; 7 Investor Mining and Tailings Safety Initiative and Council On Ethics for the Swedish National Pension Funds, Gothenburg, Sweden

Correction to: *Scientific Reports* 10.1038/s41598-021-84897-0, published online 05 March 2021

The Supplementary Information 1 file published with this Article contained an error where the Supplementary References section was omitted. The original Supplementary Information 1 file is provided below

This error has now been corrected in the Supplementary Information 1 file that accompanies the original Article.

## Supplementary Information

Supplementary Information 1.

