# Supplementary material for: Author Correction: Tailings facility disclosures reveal stability risks
Source: Sci Rep. 2021 Jun 3;11:12142. doi: 10.1038/s41598-021-91384-z (PMC8175709; doi:10.1038/s41598-021-91384-z)
Supplement: Supplementary file 1 — Supplementary Information 1. [file 41598_2021_91384_MOESM1_ESM.docx]

Supplementary Materials

**Tailings Facility Disclosures Reveal Stability Risks**

Daniel M. Franks^1^*, Martin Stringer^2^, Luis A. Torres-Cruz^3^, Elaine Baker^4,5^, Rick Valenta^2^, Kristina Thygesen^5^, Adam Matthews^6^, John Howchin^7^, Stephen Barrie^6^.

^1^Sustainable Minerals Institute, The University of Queensland, Brisbane, 4072, Queensland, Australia.

^2^W.H. Bryan Mining & Geology Research Centre, Sustainable Minerals Institute, The University of Queensland, 40 Isles Road Indooroopilly, 4068, Queensland, Australia.

^3^School of Civil and Environmental Engineering, University of the Witwatersrand, 1 Jan Smuts Avenue, Braamfontein, 2000, Johannesburg, South Africa.

^4^UNESCO Chair in Marine Science, The University of Sydney, Madsen FO9, 2006, New South Wales, Australia.

^5^GRID Arendal, P.O. Box 183, N-4802, Arendal, Norway.

^6^Investor Mining and Tailings Safety Initiative & Church of England Pensions Board, Church House, Great Smith Street, London, SW1P 3AZ, United Kingdom.

^7^Investor Mining and Tailings Safety Initiative & Council on Ethics for the Swedish National Pension Funds, Sweden

*Correspondence to: d.franks@uq.edu.au.

Materials and Methods

Supplementary Text

Figs. S1 to S11

Tables S1 to S4

Data S1

Materials and Methods

Survey of Tailings Facilities

On April 5, 2019, the Church of England (CoE) Pensions Board and the Council on Ethics of the Swedish National Pension Funds, on behalf of 112 investors, representing US$14 trillion in assets under management (the Investor Mining and Tailings Safety Initiative), wrote to Board Chairs and Chief Executive Officers of listed extractive companies and requested specific disclosure on tailings facilities (*4*). The disclosure questions were developed in consultation with independent technical advisors, industry experts, and four mining companies. Table S1 provides a full list of the disclosure questions. The letter requested that the responses be uploaded to the company website, signed by the CEO or Board Chair within 45 days. A follow-up letter was sent on April 17, setting an extended deadline of June 7, 2019 for the disclosure (*21*). Correspondence was sent to a total of 727 companies, representing publicly listed mining, as well as oil and gas companies. The later were included due to the potential exposure to tailings from oil sands operations and joint ventures.

A specialist Environmental, Social and Governance (ESG) financial services provider was commissioned to compile the list of companies and distribute the letter requesting disclosure. The list of companies was generated using the Global Industry Classification Standard. Companies in the following sub-industry codes were contacted: oil and gas drilling, oil and gas exploration and production, integrated oil and gas, coal and consumable fuels, fertilizers and agricultural chemicals, aluminium, diversified metals and mining, copper, gold, precious metals and minerals, silver, steel, and construction materials. An additional 88 small and mid-market companies not listed in the above codes were added by investor participants in the initiative.

The request specified that companies should report all tailings facilities where the company has any interest, through subsidiaries, partnerships, joint ventures both incorporated and unincorporated, and any other enterprises of whatever legal form. All joint venture partners were requested to report on jointly owned facilities, even if the reporting company was not the operating partner.

Of the 727 companies contacted:

- 339 responded (representing 47 per cent of the companies contacted)
- 187 of these companies confirmed they did not have tailings facilities (representing 55 per cent of those responding and 26 per cent of all companies)
- 152 confirmed they did have tailings facilities.

As of March 2020, 45 of the companies that confirmed exposure to tailings facilities had not published their disclosure on a website or asked for extra time to complete their disclosure.

For the mining sector specifically:

- 45 out of the 50 largest mining companies by market capitalisation in the world responded
- 83 per cent of the industry by market capitalisation responded
- 60 per cent of the industry by market capitalisation publicly disclosed
- All 23 out of 23 publicly listed members of the ICMM publicly disclosed.

The proportion of market capitalisation of the respondents was calculated on 4 November 2019 using the Thomson Reuters Eikon financial data platform.

Data limitations

The method used to request information disclosure on tailings facilities from publicly-listed contemporary companies has produced a dataset that is likely more representative of active tailings facilities, omitting some closed facilities and the large number of abandoned facilities for which there is no longer an owner responsible. There is also a possibility that the survey under-samples less diligent companies, with lower governance standards, who failed to respond to the disclosure request.

The dataset does not include information from companies that are not publicly listed, such as state-owned entities and privately-owned companies, including many mid-sized and junior companies. This contributes to an under-representation of facilities in countries were there the number of state-owned and privately-owned mining operations is significant (e.g. China, India and Chile), and potentially an over-representation of larger facilities.

There are incentives for companies to under-report on parameters such as the existence of stability issues, and to that extent the analysis and data presented herein should be considered conservative. There is evidence in the disclosures that some companies chose to affirmatively answer the question about the presence of a stability issue only when the issue in question still affects the current state of the facility. The failure of tailings facilities also has the effect of contributing to under-reporting by the very fact that in some cases those facilities no longer exist and thus their characteristics are not disclosed.

Another limitation of the dataset is that the occurrence of multiple instances of stability issues at the same facility is not recorded. This may have the effect of undercounting the prevalence of stability issues for facilities prone to experiencing them. Due to this limitation, the findings on stability are not a calculation of the rate of instability over a normalised period of time; however, they do enable the comparison of general stability trends between facility types.

Data collation and validation

With funding support from the United Nations Environment Program (UNEP) and the Investor Mining and Tailings Safety Initiative, GRID-Arendal compiled the data into a database for analysis. The individual company disclosures were compiled independently by two additional research teams from The University of Queensland and University of the Witwatersrand, and shared with the GRID-Arendal team for cross-checking, comparison and data-cleaning. A searchable online database of the disclosures was published by GRID-Arendal on the 24^th^ of January 2020, as the Global Tailings Portal (<http://tailing.grida.no>).

In December of 2019 and January of 2020, a compilation of the disclosed data was sent to each reporting company for verification. The majority of disclosing companies responded to this extra request, resulting in 86 per cent of the entries of the dataset being subject to this additional layer of verification. A full list of the companies that were contacted and the status of their disclosure is publicly available and published on the Investor Mining and Tailings Safety Initiative website (*22*). The version of the dataset analysed in this paper was current as at February 26, 2020.

Due to duplicate reporting by multiple owners, the disclosures were corrected for analysis to represent only unique tailings facilities. Where there were discrepancies in the reported data by multiple owners of the same facility, we prioritised data for analysis which were disclosed by the operating companies. Where the ownership of the facility was a separate joint-venture company, we prioritised the data reported by the owner with the highest ownership share. In the case of 50/50 joint ventures, we prioritised the data of the owner by alphabetical order.

Each “tailings facility” in the dataset represents a unique tailings structure. In some cases, tailings facilities may consist of multiple structures. This generated a second type of duplicate in the raw data that is relevant for calculations of volume. Companies that reported facilities with multiple structures sometimes reported the same total volume and planned volume for multiple data entries. In our calculations of volume, duplicate data were corrected by evenly distributing the reported volume against the number of structures that make up the facility. It is also worth noting that “tailings facilities” in the dataset include tailings production at mines, but also tailings, slimes, ash and other wastes produced at mineral processing and smelting facilities.

Analysis methods

The S&P Global Metals and Mining Industry database was used to assign individual mine site mineral production to the active tailings facility entries. The most recent S&P Global production figures (2018) were used (<https://platform.mi.spglobal.com>).^[[1]](#footnote-1)^ Global mineral production figures and mean annual prices from the United States Geological Survey (USGS), *Mineral Commodity Summaries* (*23*; reporting 2018 data) were used to calculate the representativeness of the dataset as a function of global production and to project a global estimate of tailings production and number of facilities. USGS commodity summaries do not include artisanal and small-scale mining production, for which extraction is commonly of placer deposits with consequent low production of tails. The tailings facility dataset represents an average of 36% per cent of global commodity production. The relatively high sample rate provides confidence in the representativeness of the dataset for publicly-owned active tailings facilities.

The expected tailings production (as stored in tailings facilities) for each mine was calculated by using the annual average of the planned tailings storage in five-years, which was reported by the companies. Production data are available in the S&P database for a range of commodities (bauxite, coal, cobalt, copper, diamonds, gold, iron ore, lanthanides, lead, lithium, molybdenum, nickel, niobium, palladium, phosphate, platinum, potash, silver, tin, uranium, zinc). The number of active facilities attributed to each of these commodities was assigned according to the value share of their production in each mine. For each commodity, *i*, with annual production, P*_i_*, and mean price for that production year, $*_i_*, the number of active facilities attributed to that commodity, N*_i_*, is calculated by:

$$N_{i}= \sum_{all mines, m} N_{m}\frac{{\$}_{i}P_{i,m}}{\sum_{j} {\$}_{j}P_{j,m}}$$

The sum over *j* includes all the commodities being produced by that mine (including *i*), and N*_m_* is the number of active facilities at each mine, *m*. This number is then scaled up to a world estimate using the USGS world production for that commodity.

$$N_{i,world}=\frac{P_{i,world}}{\sum_{m} P_{i,m}}N_{i}$$

Planned storage increase was attributed and scaled in the same way.

The world estimates for active facilities were on average 7.4 times the database totals. For increased storage volume this scaling was 11.9. For commodities where production data are not available from the S&P Global database (alumina, aluminium, borates, chromite, ferrochrome, ferromanganese, ferrovanadium, ilmenite, manganese, rutile, tantalum, titanium, vanadium, oil sands, refineries, smelters, power plants; representing 12 per cent of the reported active facilities), these average scalings were used to translate the database totals to global estimates. Data for the above calculations are shown in Table S2.

Tailings production by weight were calculated assuming the modal average of tailings bulk density reported by 20 companies as part of the disclosures (1.3 *t*/m^3^). The reprocessing and reclamation of tailings (a type of negative production of tailings) was not considered in the calculation of expected future tailings production.

The number of active tailings facilities was estimated by projecting the proportion of global production represented by the mines in the tailings facility dataset for active mines. If the proportion of active, inactive, and closed facilities in the database is assumed to remain constant among facilities that are not in the database, the total number of facilities, active, inactive and closed, can also be estimated.

For data analysis purposes Modified Centreline facilities were categorized together with Centreline facilities. Facilities where waste rock and tailings where co-disposed were categorized as Other. Operations that produce paste or thickened tailings were classified by companies by the facility raise type, rather than whether the tailings themselves have been dewatered. A small number of Central Thickened Discharge facilities were reported in the dataset, but not enough to undertake meaningful analysis. These facilities were also categorized as Other.

Data on seismic hazard were derived from the Global Seismic Hazard Assessment Program (*24*) which provides a global dataset of seismic risk based on Peak Ground Acceleration risk estimates. Data on wind were sourced from (*25*), and data on precipitation sourced from (*26*).

Supplementary Text

Stability of Tailings Facilities – Age

All other things being equal, we would expect older structures to be more likely to have reported a stability issue than younger structures. This is because older facilities have had a longer opportunity for a stability issue to manifest. To control for this, we mapped the number of facilities that had reported a stability issue against the age of the facility in years. This was done for all active facilities, and for all active upstream, downstream and dry-stack facilities specifically. The results are presented in Fig. S6, which shows the number of facilities reporting a stability issue, by facility age and the proportion of facilities of different ages that had reported a stability issue.

As to be expected, a higher proportion of long-active conventional tailings facilities reported a stability issue. Upstream facilities demonstrate a relatively higher prevalence of stability issues just ten to twenty years after construction. The very small number of active dry-stack facilities reporting a stability issue (1) produces an artefact of apparently high proportion of stability concerns at facilities aged 40-50 years old, due to this being the age of the single active dry-stack facility with a stability issue.

Stability of Tailings Facilities – Embankment Height

The dataset also points to a relationship between facility embankment height and whether a facility had reported a stability issue, but this relationship is not straightforward (Fig. S7). The likelihood of a stability issue being reported for a facility with an embankment between 80-100m is notably 5 times higher than for facilities with embankments between 0-20m. But in the relatively small number of cases where an embankment height exceeds 100m, there is a decline in the proportion of facilities that reported a stability issue. A possible explanation for this, may be that higher standards of construction have been applied for facilities with very high embankments (although we have no direct measure of this).

Stability of Tailings Facilities – Volume

The larger the facility, the more likely it is to have reported a stability issue (Fig. S8). Due to the very large range of reported volumes, from just 10m^3^ to over 1 billion m^3^, a logarithmic scale is used to display the distribution. The broad trend in stability issues that this reveals should be interpreted accordingly: similar *proportional* increases in volume (e.g. 10 times greater) seem to be associated with similar *absolute* increases in the fraction with issues (e.g. 5% higher). This analysis cannot distinguish between the possibility that the increased incidence is due to the greater surface area of the material, the greater stress from the increased mass, or the potential for these or other factors (such as age) to act in combination.

Stability of Tailings Facilities – Seismic Hazard

Facilities built in seismically active regions might be expected to show a higher incidence of stability issues. Figure S9 shows the distribution of tailings facilities by seismic hazard and the proportion of tailings facilities with a stability issue by seismic hazard.

Most facilities are built in locations with a seismic hazard below 1. As seismic hazard increases, the likelihood of a facility having reported a stability issue initially decreases. However, above a seismic hazard of three, the proportion of facilities reporting a stability issue then increases. This relationship is not attributable to other factors that may be changing coincidentally with seismic hazard. In particular, facility height and storage volume do not change significantly for any given range in seismic hazard.

It is worth noting that the proportion of upstream facilities is lower in seismically active regions, with a corresponding increase in downstream facilities (Fig. S9). This may be due to concerns by governments and companies about the relative stability of the upstream raise type and may be a factor in the lower likelihood of a reported stability issue with increasing seismic hazard (between 0-3). Another possible interpretation for the described trend (though one for which we do not have direct data), is that facilities in locations with elevated seismic hazard may be built to higher standards of construction than facilities in locations with very low seismic hazard, thus leading to an initial improvement in geotechnical stability with increasing seismic hazard. However, above a certain point of seismic hazard (3+), facility stability may be reduced even for those facilities built to higher construction standards.

Stability of Tailings Facilities – Statistical Analysis

As discussed above, stability instances correlate with several properties, and these correlations should ideally be taken into account in order to interpret the results of Fig. 3. Each raise type sample has a different distribution of these properties, and we can test whether this distribution could account for the difference in the stability issues.

The null hypothesis for this test is that only these other variables influence stability issues, not raise type. If this hypothesis were true, and we took any two subsamples from the dataset which had almost identical distributions in these variables, we would expect to find almost the same stability fraction in both subsamples. This expectation would hold even if one sample is comprised entirely of facilities with a given raise type, and the other contains none.

To test the hypothesis, we compared each raise type sample, the ‘test’ sample, with 100 different ‘mock’ subsamples that were created by resampling from the control group (the facilities with all other raise types that have finite values for all the variables we are controlling for). The subsamples were selected to mimic the distributions of variables of the test sample, incorporating some randomization in the selection process. The results of this test are shown in Fig. S10 (all facilities) and S11 (active facilities).

**Fig. S1.** Current volume of tailings under storage (in cubic kilometres) by raise type of active, inactive and closed facilities. *Note*: shading indicates active facilities.


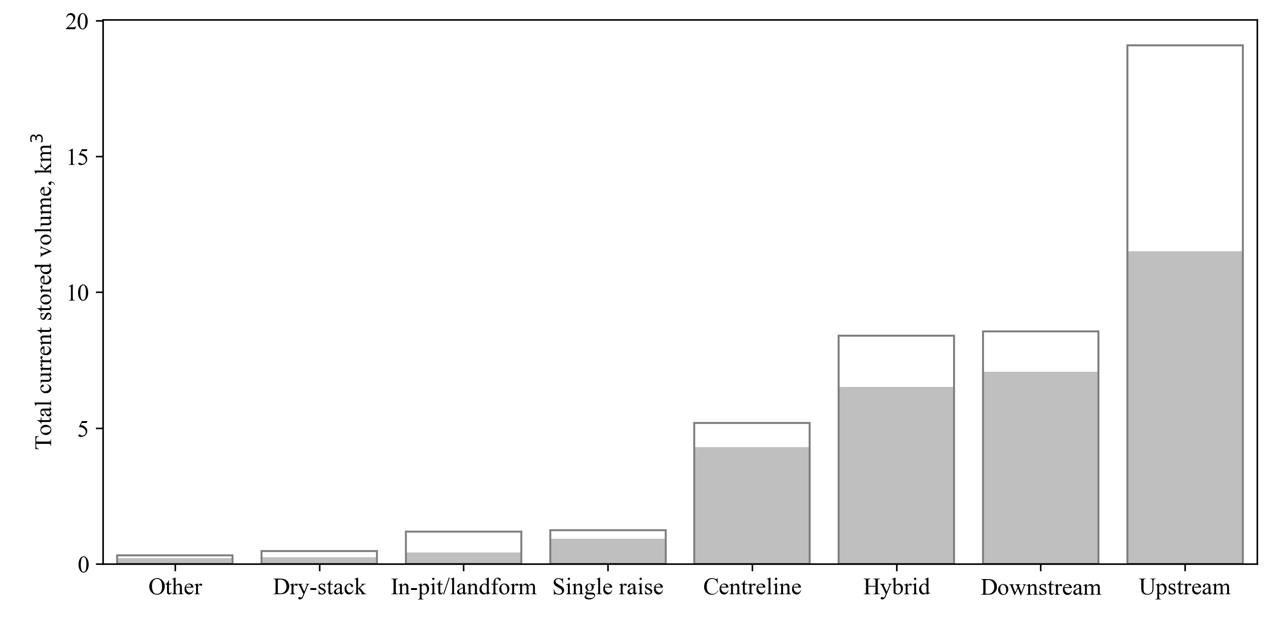


**Fig**. **S2.** Tailings facilities by decade of construction. *Note*: shading indicates active facilities.


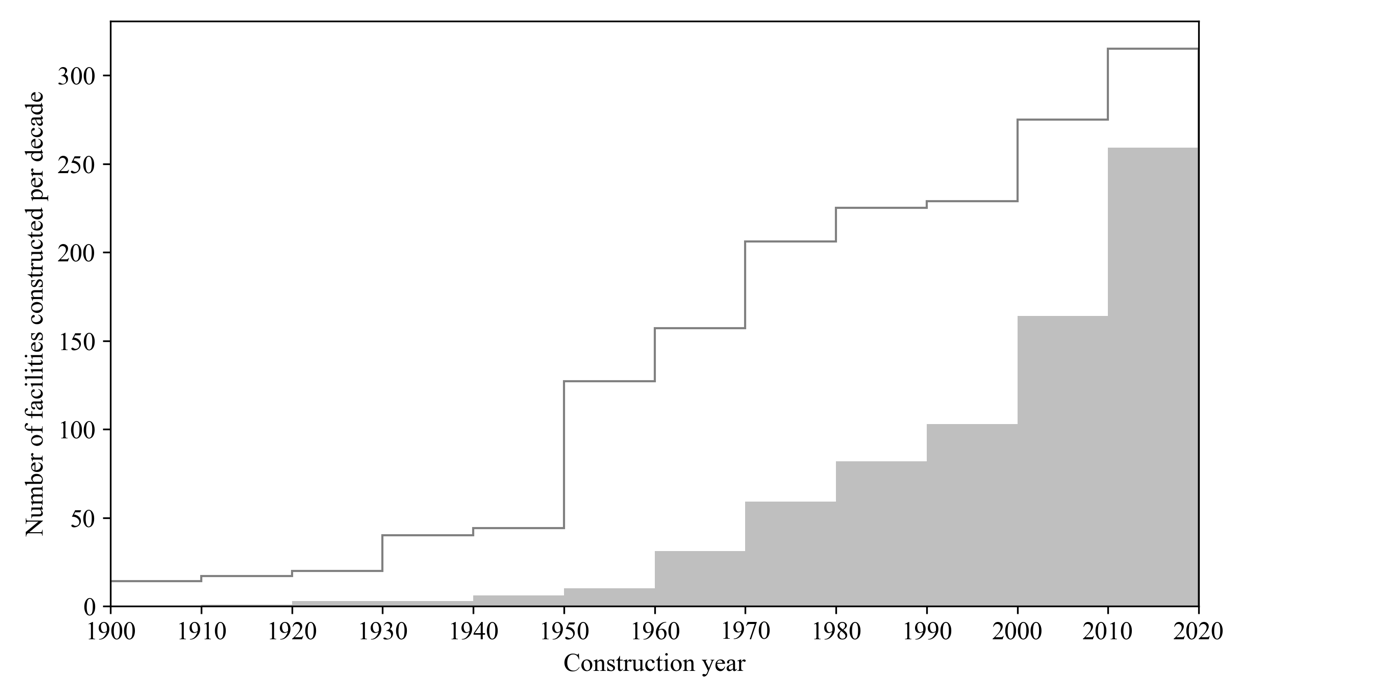


**Fig. S3.** Distribution of tailings facility raise type by continent. *Note*: shading indicates active facilities; countries are assigned to continents according to <https://www.geonames.org/countries/>

*
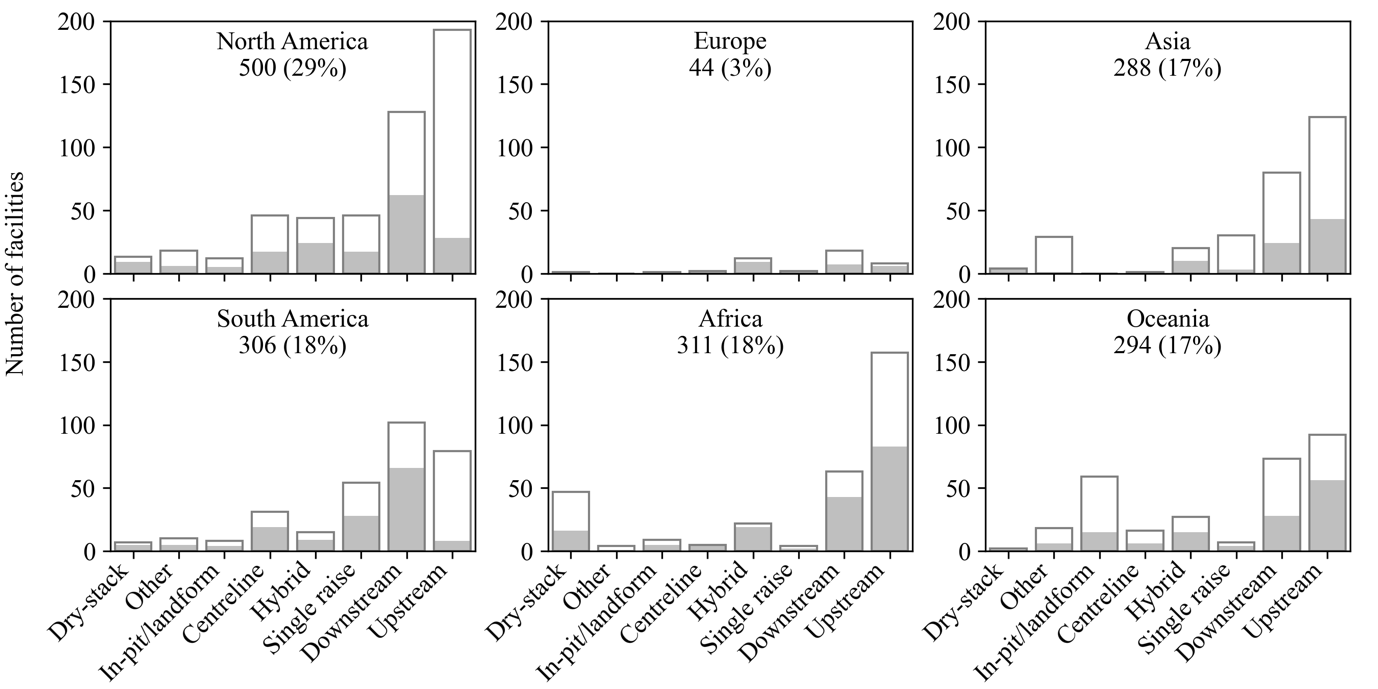
*

**Fig*.* S4.** Proportion of facility raise type by seismic hazard.

*
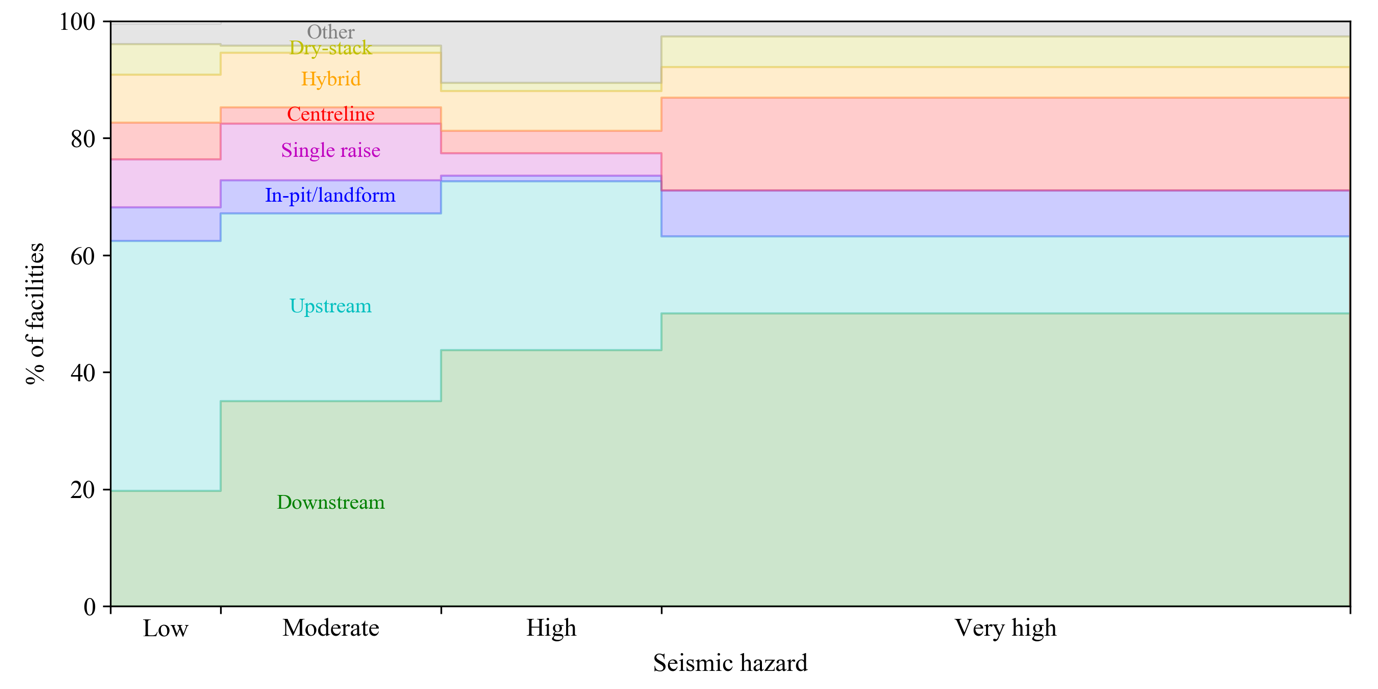
*

**Fig. S5.** Dry-stack facilities by decade of construction. Top: number of facilities. Bottom: proportion of facilities.


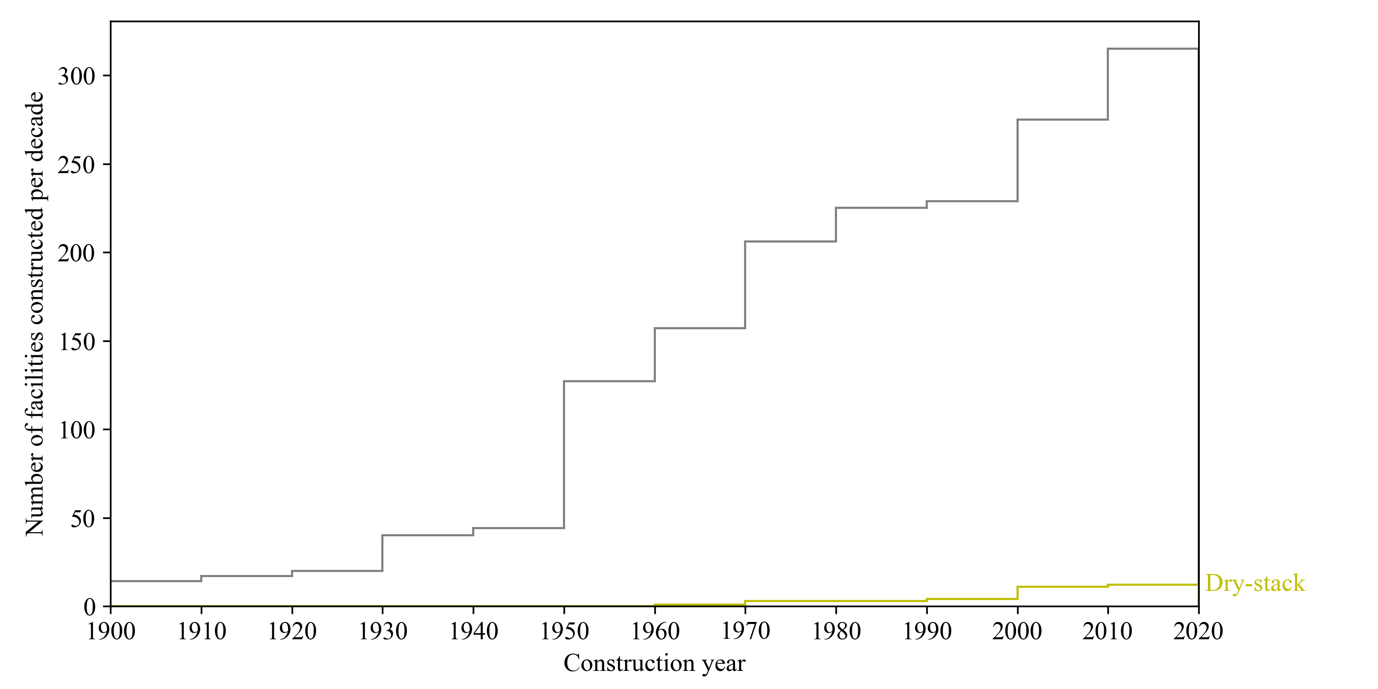

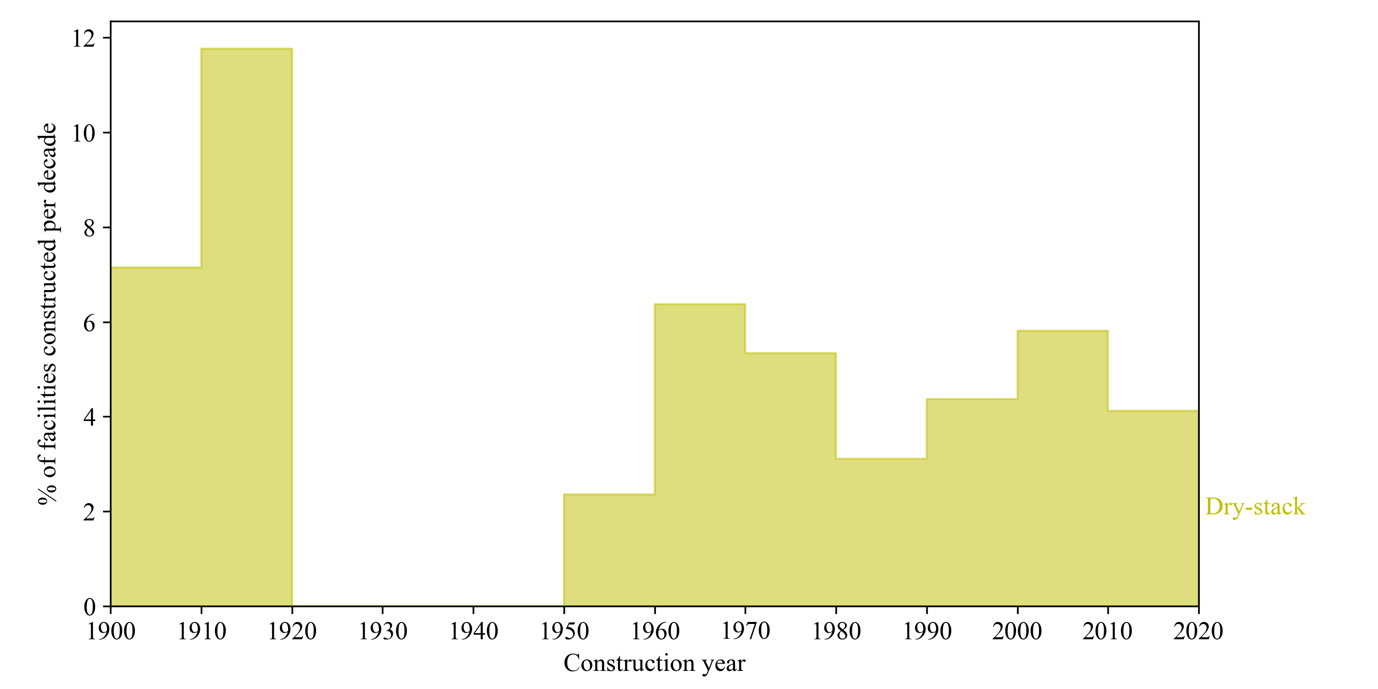


**Fig. S6.** Relationship between facility age, facility raise type and stability issue. Top: number of active facilities reporting a stability issue. Bottom: proportion of active facilities in each age category reporting a stability issue.

**
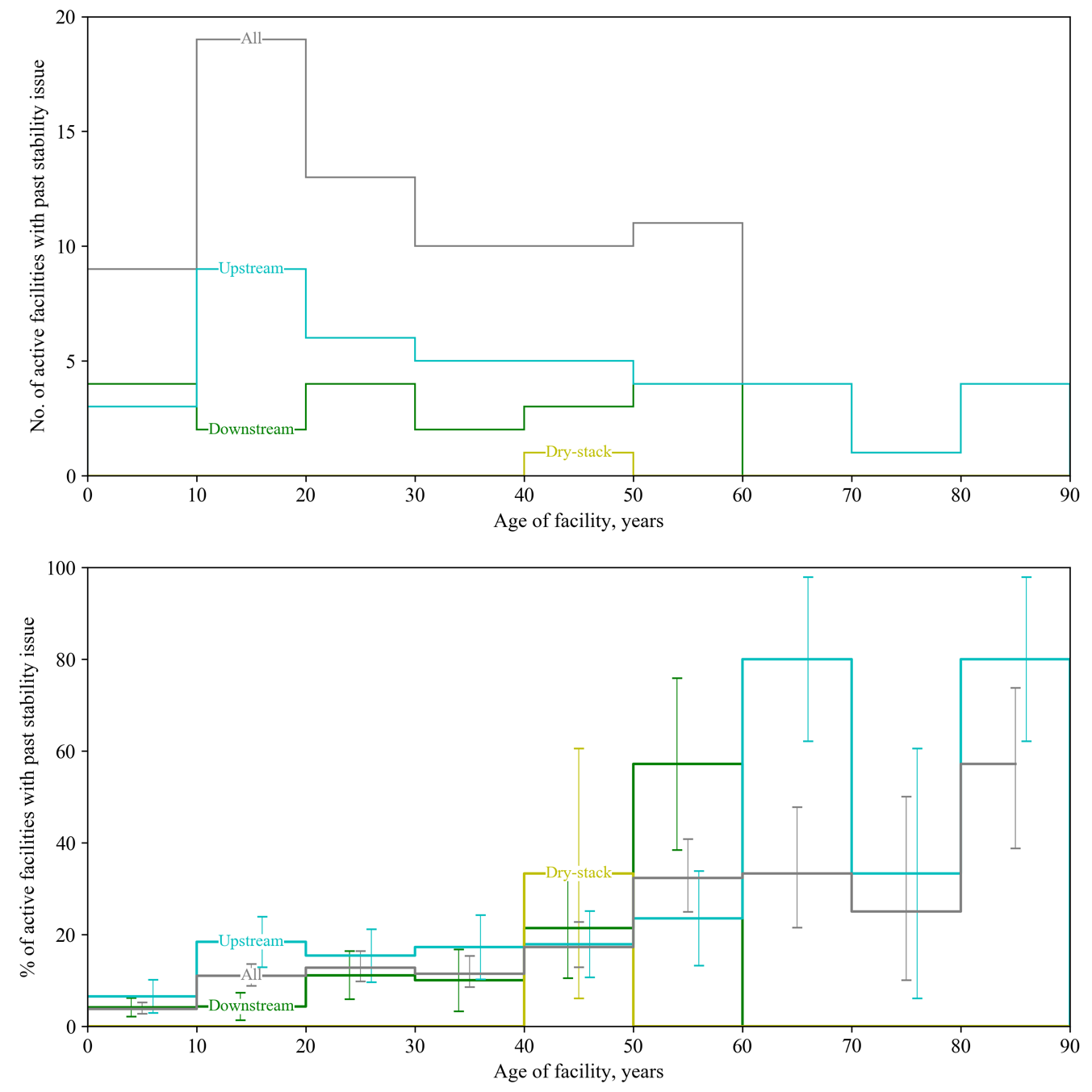
**

**Fig. S7.** Relationship between facility embankment height and reported occurrence of stability issues, all facilities. Top: distribution of tailings facilities by embankment height. Bottom: proportion of facilities reporting a stability issue by embankment height. *Note*: shading indicates number of facilities reporting a stability issue; there are no instances of stability issues in heights above 140m; vertical error bars for these categories show the range of fractions for which the probability of finding zero in a sample of that size is greater than 74% (the same confidence interval as shown for the other points).


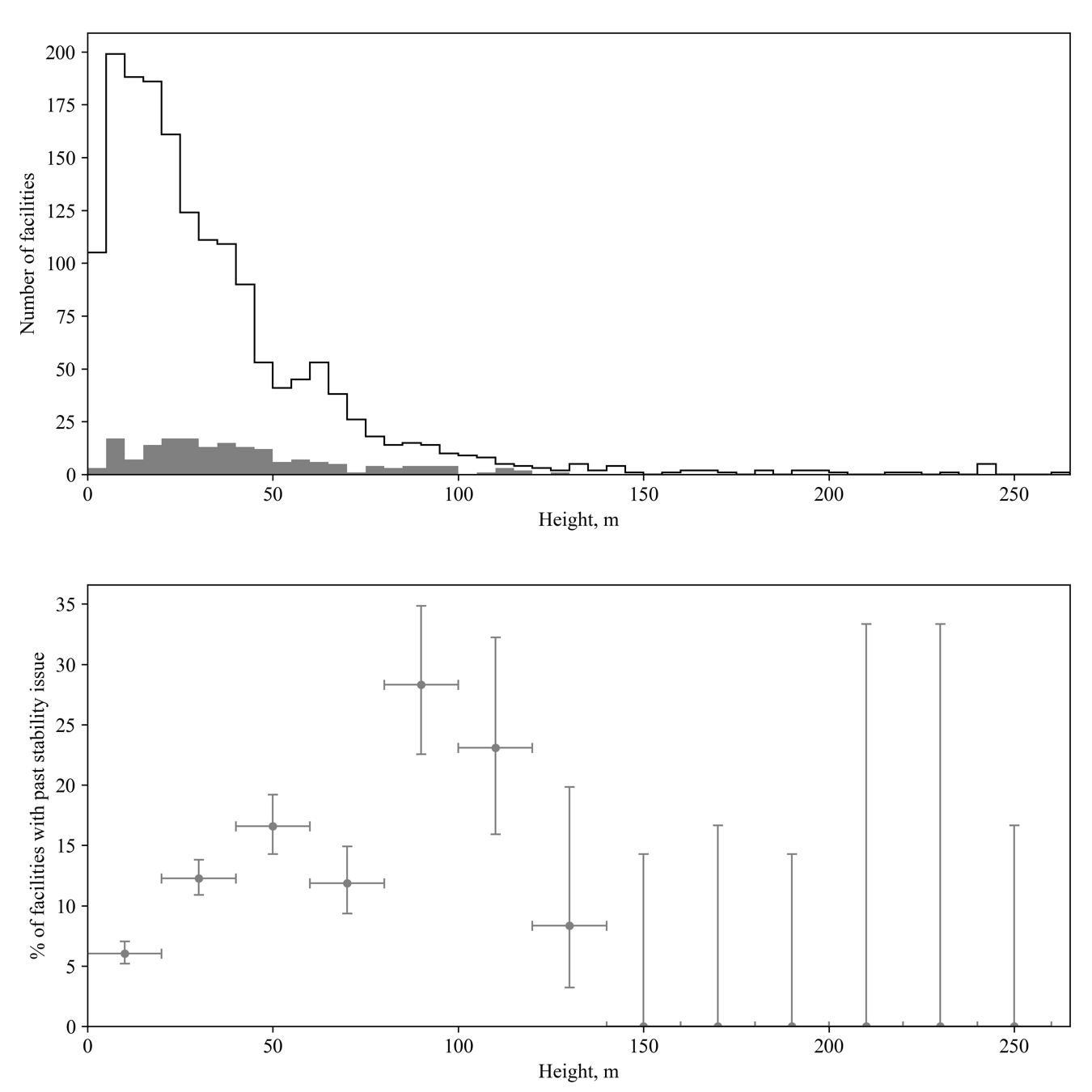


**Fig. S8.** Relationship between facility volume and reported stability issue, all facilities. Top: distribution of tailings facilities by volume. Bottom: proportion of facilities reporting a stability issue by facility volume. *Note*: shading indicates number of facilities reporting a stability issue.


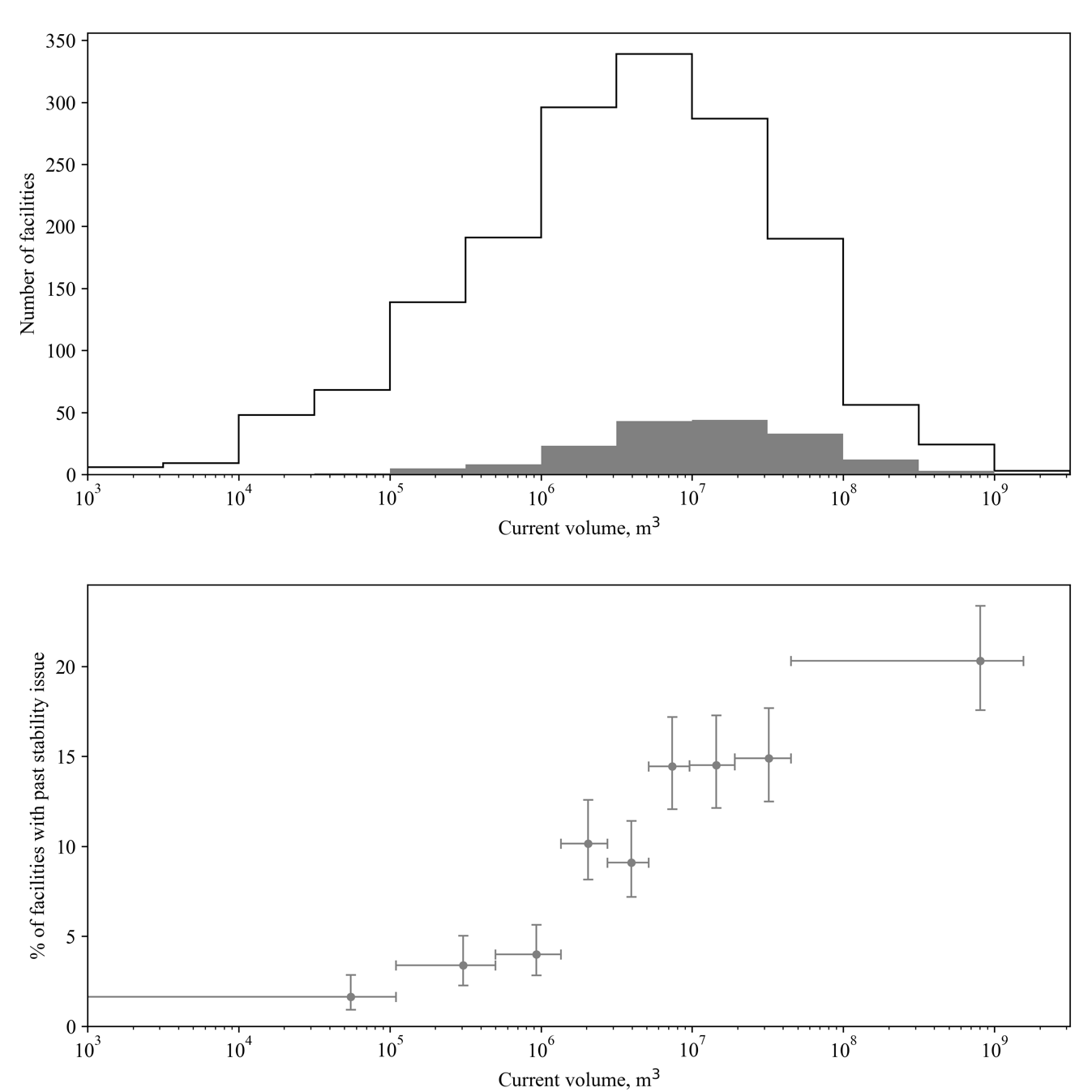


**Fig. S9.** Relationship between seismic hazard and history of stability issue, all facilities. Top: distribution of tailings facilities by seismic hazard. Bottom: proportion of stability issue by seismic hazard. *Note*: shading indicates number of facilities reporting a stability issue; seismic hazard ratings correspond to those defined by the Global Seismic Hazard Assessment program.

**
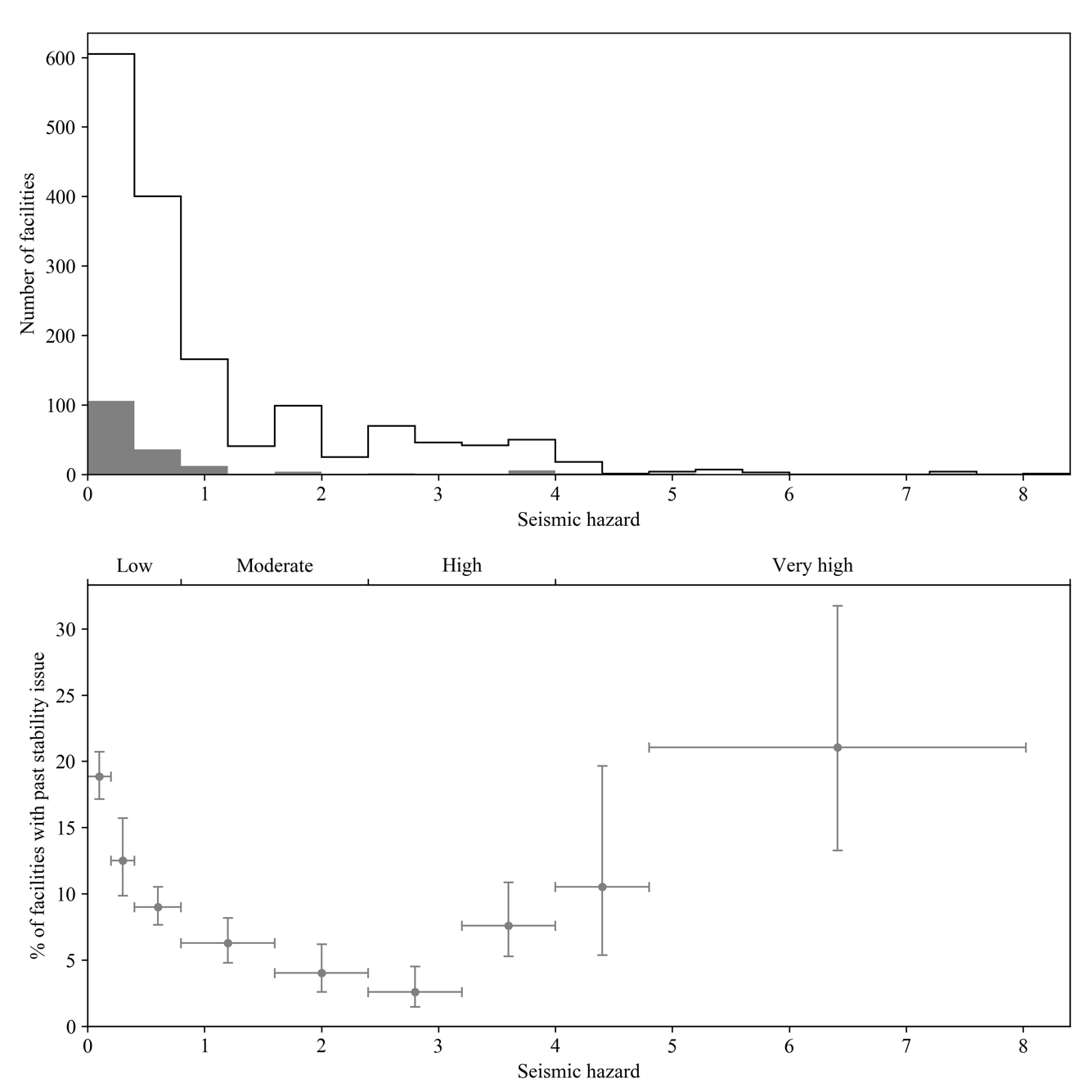
**

**Fig. S10 (below).** Analysis of the effect of bias on samples of different raise type (see text). **Right hand panels** show the distribution of subsamples across the six quantitative variables that we are controlling for. Blue line shows the raise type sample, pink lines show the 100 different mock subsamples, and grey shows the control sample of all other raise types. **Left panels** compare the mean instance of stability issues in these samples, using the same colors. The difference between the control sample and the mock subsamples, shown as a grey arrow, provides an estimate of the effect that the distribution of properties for that raise type would be expected to have on the instance of stability issues. Pink bars show the distribution of stability instances for the mock samples.

**
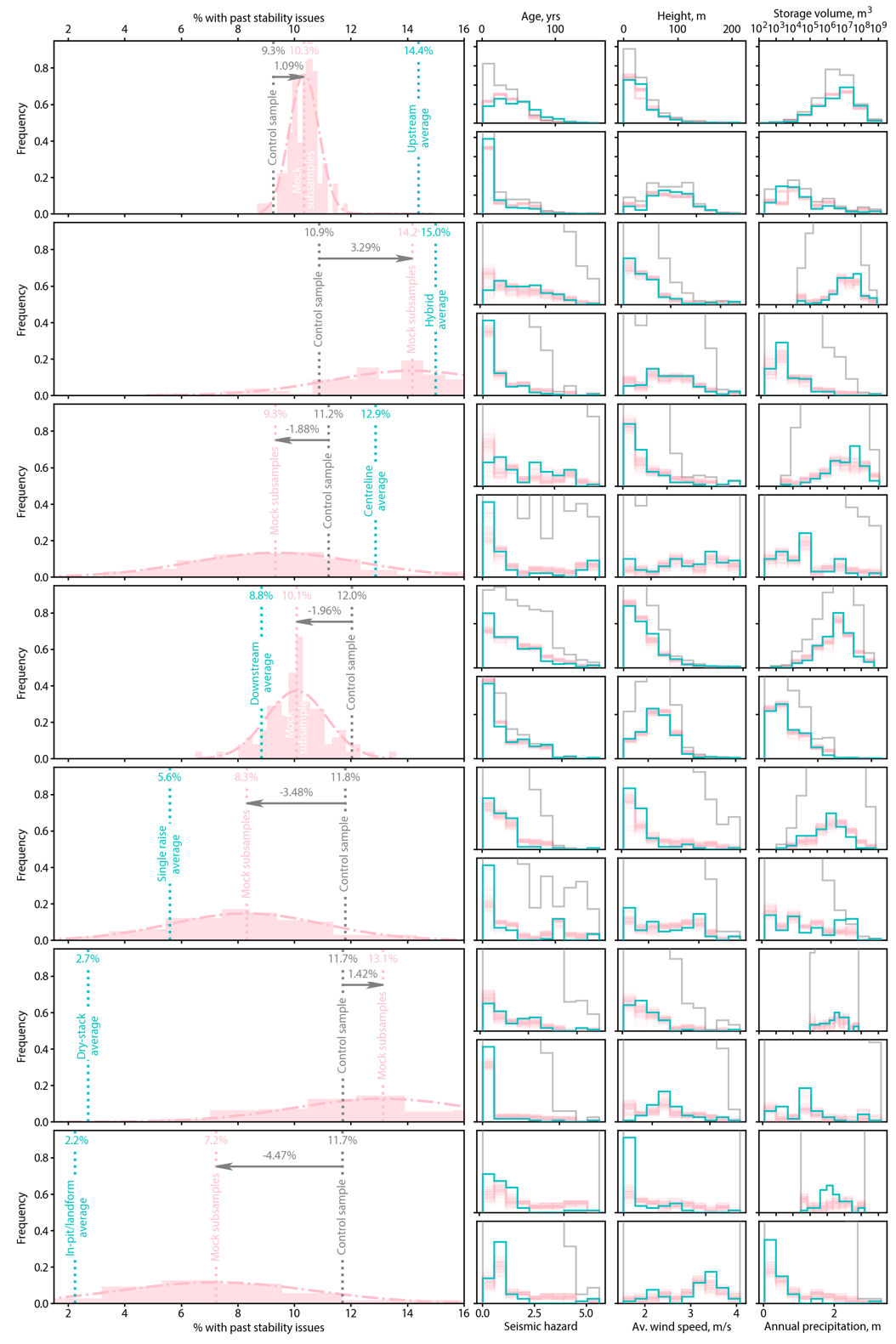
**

**Fig. S11.** As Fig. S10, but including only active facilities.


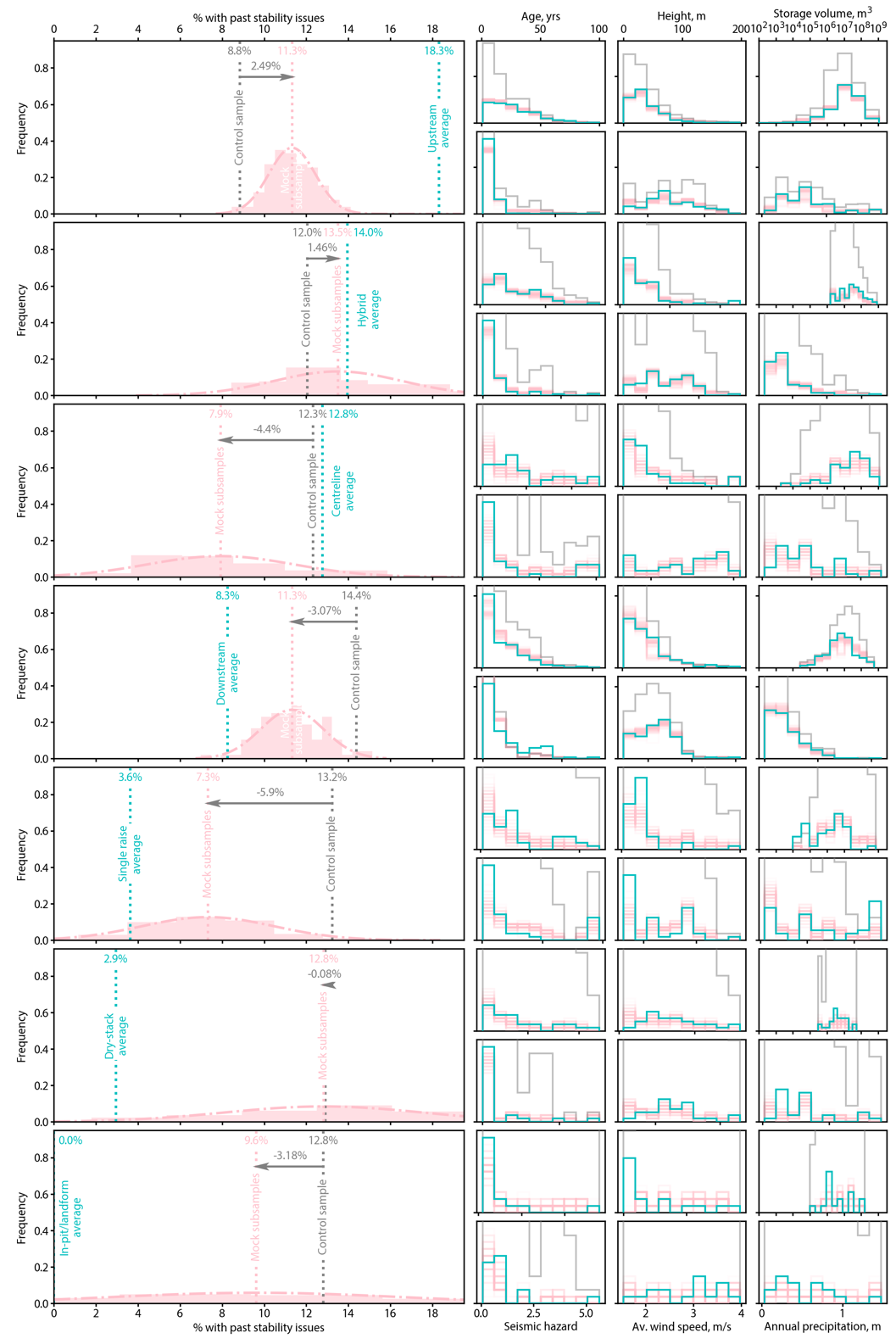


**Table S1**. Questions included in the global survey of tailings facilities.

| 1. "Tailings Facility" Name/identifier | Please identify every tailings storage facility and identify if there are multiple dams (saddle or secondary dams) within that facility. Please provide details of these within question 20. |
| --- | --- |
| 2. Location | Please provide Long/Lat coordinates |
| 3. Ownership | Please specify: Owned and Operated, Subsidiary, JV, NOJV, as of March 2019 |
| 4. Status | Please specify: Active, Inactive/Care and Maintenance, Closed etc. |
|  | We take closed to mean: a closure plan was developed and approved by the relevant local government agency, and key stakeholders were involved in its development; closed facility means the noted approved closure plan was fully implemented or the closure plan is in the process of being implemented. A facility that is inactive or under C&M is not considered closed until such time a closure plan has been implemented. |
| 5. Date of initial operation | (date) |
| 6. Is the Dam currently operated or closed as per currently approved design? | Yes/No. If 'No', more information can be provided in the answer to Q20 |
| 7. Raising method | Note: Upstream, Centerline, Modified Centreline, Downstream, Landform, Other. |
| 8. Current Maximum Height | Note: Please disclose in metres |
| 9. Current Tailings Storage Impoundment Volume | Note: (m3 as of March 2019) |
| 10. Planned Tailings Storage Impoundment Volume in 5-years-time. | (m3 as planned for January 2024) |
| 11.Most recent Independent Expert Review | (date) For this question we take ‘Independent’ to mean a suitably qualified individual or team, external to the Operation, that does not direct the design or construction work for that facility. |
| 12. Do you have full and complete relevant engineering records including design, construction, operation, maintenance, and/or closure? | (Yes or No) We take the word “relevant” here to mean that you have all necessary documents to make an informed and substantiated decision on the safety of the dam, be it an old facility, or an acquisition, or legacy site. More information can be provided in your answer to Q20 |
| 13. What is your hazard categorisation of this facility, based on the consequence of failure? |  |
| 14. What guideline do you follow for the classification system? |  |
| 15. Has this facility, at any point in its history, failed to be confirmed or certified as stable, or experienced notable stability concerns, as identified by an independent engineer (even if later certified as stable by the same or a different firm). | (Yes or No) We note that this will depend on factors including local legislation that are not necessarily tied to best practice. As such, and because remedial action may have been taken, a “Yes” answer may not indicate heightened risk.  Stability concerns might include toe seepage, dam movement, overtopping, spillway failure, piping etc. If yes, have appropriately designed and reviewed mitigation actions been implemented?  We also note that this question does not bear upon the appropriateness of the criteria, but rather the stewardship levels of the facility or the dam. Additional comments/information may be supplied in your answer to  Q20. |
| 16. Do you have internal/in house engineering specialist oversight of this facility? Or do you have external engineering support for this purpose? | Note: Answers may be "Both". |
| 17. Has a formal analysis of the downstream impact on communities, ecosystems and critical infrastructure in the event of catastrophic failure been undertaken and to reflect final conditions? If so, when did this assessment take place? | Note: Please answer 'yes' or 'no', and if 'yes', provide a date. |
| 18. Is there a) a closure plan in place for this dam, and b) does it include long term monitoring? | Please answer both parts of this question (e.g. Yes and Yes) |
| 19. Have you, or do you plan to assess your tailings facilities against the impact of more regular extreme weather events as a result of climate change, e.g. over the next two years? | (Yes or No) |
| 20. Any other relevant information and supporting documentation. | Note: this may include links to annual report disclosures, further information in the public domain, guidelines or reports etc. |

**Table S2**. Estimate of global tailings storage volume increase per year and number of active, inactive and closed tailings facilities.

**Table S3**. Occurrence of a stability issue by raise type and governance context.

| **Raise Type** | **All facilities** | **Active-only facilities** | **OECD countries (active-only)** | **Non-OECD countries (active-only)** | **ICMM member (active-only)** | **Non-ICMM member (active-only)** |
| --- | --- | --- | --- | --- | --- | --- |
| Upstream | 94 of 653 (14.4%) | 41 of 224 (18.3%) | 12 of 87 (13.8%) | 29 of 137 (21.2%) | 24 of 142 (16.9%) | 17 of 82 (20.7%) |
| Downstream | 41 of 464 (8.8%) | 19 of 230 (8.3%) | 7 of 106 (6.6%) | 12 of 124 (9.7%) | 8 of 128 (6.2%) | 11 of 102 (10.8%) |
| Hybrid | 21 of 140 (15.0%) | 12 of 86 (14.0%) | 7 of 46 (15.2%) | 5 of 40 (12.5%) | 4 of 34 (11.8%) | 8 of 52 (15.4%) |
| Centreline | 13 of 101 (12.9%) | 6 of 47 (12.8%) | 2 of 25 (8.0%) | 4 of 22 (18.2%) | 3 of 31 (9.7%) | 3 of 16 (18.8%) |
| Single raise | 8 of 143 (5.6%) | 2 of 55 (3.6%) | 2 of 22 (9.1%) | 0 of 33 (0.0%) | 0 of 40 (0.0%) | 2 of 15 (13.3%) |
| In-pit/landform | 2 of 89 (2.2%) | 0 of 30 (0.0%) | 0 of 20 (0.0%) | 0 of 10 (0.0%) | 0 of 17 (0.0%) | 0 of 13 (0.0%) |
| Dry-stack | 2 of 74 (2.7%) | 1 of 34 (2.9%) | 0 of 10 (0.0%) | 1 of 24 (4.2%) | 1 of 25 (4.0%) | 0 of 9 (0.0%) |
| Other | 1 of 79 (1.3%) | 0 of 19 (0.0%) | 0 of 12(0.0%) | 0 of 7 (0.0%) | 0 of 12 (0.0%) | 0 of 7 (0.0%) |

**Table S4**. Five most common consequence classification schemes reported against in the dataset. A small number of facilities reported against more than one scheme.

| **Name** | **Number (all facilities)** | **Number (active facilities)** |
| --- | --- | --- |
| Canadian Dam Association (CDA) | 577 (33.1%) | 225 (31.0%) |
| Australian National Committee on Large Dams (ANCOLD) | 243 (13.9%) | 128 (17.7%) |
| South African National Standards (SANS) | 158 (9.1%) | 87 (12.0%) |
| Brazilian Ordinance 70.389/17 (BRA) | 114 (6.5%) | 63 (8.7%) |
| Anglo American Technical Standard (AA) | 98 (5.6%) | 47 (6.5%) |
| **Total** | 1190 of 1743 (68.3%) | 550 of 725 (75.9%) |

Data S1. (separate file)

Data file of global survey of tailings facilities

1. Except bauxite, where the most recent production data available in the S&P Global database were 2016. [↑](#footnote-ref-1)
